# Service provision for depressed children and youth: a survey of the scope and nature of services in Ontario

**DOI:** 10.1186/s12913-019-4784-8

**Published:** 2019-12-09

**Authors:** Priya Watson, Kamna Mehra, Lisa D. Hawke, Joanna Henderson

**Affiliations:** Department of Psychiatry, University of Toronto, Centre for Addiction and Mental Health, 80 Workman Way, Toronto, ON M6J 1H4 Canada

**Keywords:** Depression, Children, Youth, Psychotherapy, Mental health services

## Abstract

**Background:**

The worldwide prevalence of depressive disorders among children and youth has been reported in ranges from just under 3% to over 10%. In Canada, 7% of youth report past year depression, which is higher than any other age demographic. Yet, many of these youth do not receive evidence based interventions, increasing their risk for serious lifetime consequences. To better understand low service use, it is crucial to map and evaluate current services. This study aimed to determine the scope and nature of services available to depressed children and youth, and compare services to best evidence treatment guidelines.

**Methods:**

Several government and non-government resources were utilized to develop a new multi-sectoral database of depression services for children and youth across Ontario. An online survey was sent to program managers serving children/youth with depression, examining agency characteristics, populations served, services provided, patterns of service use, evaluation activities, and research priorities.

**Results:**

413 agencies with 869 program managers participated, representing mental health, addictions and other sectors. Age groups served included children up to 12 years of age (31%), adolescents aged 13–17 (70%) and transition aged youth (18–25 years) (81%). Over half of respondents worked in the mental health (43.4%) or mental health and addiction (24.4%) sectors. The most frequently provided services were assessment, psychotherapy, case management, and psychoeducation; the most common types of psychotherapy provided included cognitive behavioral therapy, social skills training, and solution-focused therapy. Psychotherapies are offered in widely varying formats, frequencies and durations. Discontinuation rates varied, with higher discontinuation among transition aged youth as compared to children. Respondents identified effective treatment, improving access, and reducing service gaps as top future research priorities.

**Conclusions:**

This study provides important new data on service provision and uptake for depressed children and youth. Comparing these results with best-evidence practice guidelines raises significant concerns about the services most commonly offered and their delivery formats. In addition, high early discontinuation rates raise questions about the service experiences of children, youth and their families. Other factors which may contribute to ongoing treatment engagement challenges include access barriers, service or client characteristics, and unintentional treatment impacts.

## Background

Major depressive disorder occurs at high rates among youth, but most will not receive professional evidence based mental health treatment for this illness. This has serious implications for the lifelong health of these youth, as depression has been shown to significantly impair social and academic development, and to be associated with other mental illness, substance use disorders, and suicide [1]. Youth report higher rates of depression than other age demographics [2], with approximately 11% reporting a lifetime prevalence of depression and 7% reporting depression in the past year [1, 3]. 5.5% of male youth and 10.7% of female youth have reported suicidal thoughts in the past year, and 4.3% youth reported having had a suicide attempt in the past year [4]. Indeed, suicide is the second leading cause of death among youth [5, 6].

Despite the high rates and severe impacts of depression, many depressed youth do not receive effective professional treatment. Fewer than a third of youth report receiving professional treatment for depression during the year of their episode [1, 3, 7]. Studies of adolescents and transition-aged youth (TAY), show that approximately 40% of depressed youth had never received professional mental health services [8]. Simultaneous with these low rates of service use, there appears to be a worsening trend for depression among youth [2, 9–11], suggesting that the number of untreated depressed youth may be growing. An increased prevalence of depression with low rates of professional treatment has serious implications for adolescent and lifelong mental health [8, 12]: in one longitudinal cohort study, adolescents with a mental disorder who did not receive professional treatment had seven times higher odds of clinical depression by age 17 than adolescents who did access services earlier in adolescence [13].

Even when adolescents enter a treatment service, many do not finish a course of treatment [14]. Understanding the factors that contribute to low rates of treatment completion is essential to effectively address this public health concern. Across studies examining engagement, factors shown to interfere with treatment engagement include caregiver/family barriers, peer relationships, client-specific characteristics, types of treatment, healthcare provider characteristics, and institutional or program variables [15, 16]. Other structural factors include low awareness of services available, deficiency of services, and a lack of time or financial resources to access services [17–20]. Social factors affecting treatment engagement include stigma and family/caregiver engagement and acceptance [15, 16, 21]. Unfortunately, interventions designed to improve youth access to services [22–25] have only a small literature evaluating their effects, with limited assessment of outcomes other than attitudinal change [21].

It is also important to consider that low rates of treatment engagement may be a response to perceptions that treatment is ineffectual or inappropriate [26]. The literature on the treatment of adolescent depression indicates high rates of drop-out and treatment non-completion among depressed youth [14, 27], raising concerns about the acceptability and effectiveness of the treatment they do receive [15]. Youth seen by mental health professionals have reported alarmingly low rates of perceived treatment effectiveness, with barely over 50% of youth in one national survey stating that the professional support they received was of some help [3]. It is unclear if the services most widely offered actually conform to best practice guidelines for evidence-based depression treatment [28] and this may lead to low service engagement or the perception of the intervention’s effectiveness by the patient.

We recently completed a systematic review of clinical guidelines for adolescent depression [29] to identify those that are most trustworthy according to international standards (AGREE II quality assessment tool [30]). Only one guideline published since 2005 was rated as ‘high quality; the NICE treatment guidelines [31, 32]. These guidelines, most recently updated in 2017, recommend both psychotherapy and pharmacological interventions for child and youth depression. For mild depression, the guidelines recommend “individual non-directive supportive therapy, group cognitive behavioral therapy (CBT) or guided self-help for a limited period (approximately 2 to 3 months) … provided by appropriately trained professionals”. Children and youth with moderate to severe depression are to be offered “a specific psychological therapy (individual CBT, interpersonal therapy, family therapy, or psychodynamic psychotherapy)” that runs for at least 3 months, and offered fluoxetine in addition to psychotherapy in severe cases where there is no response to psychotherapy alone after 4–6 weeks of treatment. Youth (aged 12–18) with moderate to severe depression may be treated at the outset with fluoxetine medication in combination with psychotherapy, or fluoxetine may be added if psychotherapy alone does not alleviate symptoms of depression [31, 33].

If children and youth are not being offered the treatments with best evidence, it may help explain the low rates of engagement and high rates of drop-out. While there is some research in adult depression documenting wide service heterogeneity and lack of adherence to guidelines [34, 35], there is less research examining whether the treatments for child and youth depression adhere to best evidence. This study contributes to the literature examining service provision for child and youth depression; to our knowledge it is the first Canadian study of its kind mapping psychosocial and other service provision for the treatment of depressed children and youth, and comparing these services to best evidence treatment guidelines.

## Methods

### Aim, design and setting

The study maps and describes the landscape of services addressing depression-related mental health concerns among children, adolescents, and TAY up to age 25 in Ontario, Canada, a province with a total population of approximately 14 million, with almost 4 million aged 24 and younger. Program managers (or their equivalent) at agencies across Ontario completed an anonymous online survey describing: the characteristics of psychosocial interventions offered at their agency (type, duration and frequency of treatments), the nature of the populations they serve (age, geographic location, comorbidities), and service use patterns such as child/youth and family engagement or discontinuation. They also reported on interagency collaboration (referrals) and evaluation activities.

### Agency database and participants

Consistent with the sampling methods of previous studies by members of the project team [36, 37], we created a comprehensive province-wide database of mental health and other cross-sectoral agencies who provide any depression-related services to children and/or youth up to 25 years of age. To our knowledge, this database is the first such comprehensive list of depression services across sectors for children and youth (including TAY) compiled in Ontario. Organizations were identified through web-based resources and governmental and other regulatory or accreditation bodies such as Children’s Mental Health Ontario (CMHO), the Ontario Network of Child and Adolescent Inpatient Psychiatry services (ONCAIPS), the Ministry of Children and Youth Services, and ConnexOntario. The database was developed through an iterative process in which we examined existing databases from government and non-governmental agencies and conducted online searches using search terms including “depression” and “mood” and noted contact information for programs for children, adolescents, youth, young adults and/or transitions-age youth (up to 25 years). We then called the identified agencies to confirm the availability of relevant services, determine the identity of the appropriate program manager and obtain their contact information, and determine other potential internal or external agencies to add to the database. We next contacted program managers to confirm program eligibility and to determine their willingness to receive study information and invitation. Eligibility criteria included: clinical mental health program for youth < 25 years, not specializing in a particular disorder other than depression, not a private fee for service program.

The resulting database consists of 1642 program managers (or equivalent) from 587 organizations providing depression-related services to children, youth and emerging adults up to age 25. The Ontario Ministry of Health website reports that there are more than 400 agencies for child and youth mental health up to 18 years of age [38]. The current study therefore represents a considerable proportion of the services and programs available.

For 1417 of the 1642 (86%) program managers identified, valid email addresses were obtained. and a REDCap [39] survey link was sent via email. The final sample consists of 869 program managers who responded to the survey with sufficient data for inclusion in the study (i.e., provided service characteristics data; 61% of eligible and reachable; 53% of all identified). These participants are from 413 agencies. For agency level analyses, responses from multiple program managers were aggregated to provide agency-level data. In a sensitivity analysis conducted using G*Power analysis software [40, 41], a two-by-two chi-squared analysis with power of .80, a significance threshold of .05, and a sample size of 413, it is possible to detect a small effect size of w = .14.

### Survey

The survey covered four domains: (1) treatment (psychosocial interventions and client characteristics); (2) service use patterns; (3) service system functioning; and (4) research priorities. This survey used a measure adapted from surveys previously developed by team members [36, 37]. Prior to launch, consultations on survey content and process were held with a Toronto-based cross-sectoral group of child and youth mental health and substance use service providers who meet monthly to share information on projects related to service system enhancement, and a national youth engagement group that focuses on youth mental health and related services.

Psychosocial interventions are those that target psychological or interpersonal factors, rather than biological factors, in the treatment of depression. These interventions include psychoeducation about depression for both the client and their caregivers, and various psychotherapies designed to reduce depressive symptoms via individual or family/parent therapies. Intervention characteristics measured included psychotherapy delivery format (e.g., group, family, individual, parent/caregiver etc.); treatment modalities (e.g., cognitive behavior therapy, interpersonal therapy, etc.); treatment duration (one time, 1–2 weeks; 1, 2, 3 or 4–5 months, 6 months to 1 year, or more than 1 year) and frequency of treatment (single session, occasionally as needed, less than weekly, weekly, more than once/week, or embedded in a day treatment or residential program), and total number of sessions (e.g., 1, 2–3, 4–8, etc.). Client characteristics included age and the estimated proportion with specific co-occurring difficulties such as anxiety, Attention Deficit Hyperactivity Disorder (ADHD), legal involvement, substance misuse, trauma, learning/neurodevelopmental issues, or relationship difficulties and social problems.

To estimate service use patterns and engagement for each of the three age groups, respondents reported on the estimated proportion of clients and their families who discontinued or were no-show to their appointments after attending one session, after 2–6 sessions, or after 7 or more sessions. System-related questions addressed whether the program made inter-agency referrals for additional services. Respondents also reported on their agency’s evaluation practices for assessing clinical outcomes and service-delivery. Finally, survey respondents described their perceived needs around future research related to services for children and youth with depression.

In order to contextualize responses and describe the sample, the survey elicited characteristics of each agency (geographic region, agency size), program (sector, age groups served, types of services offered for each age group), and respondent characteristics, including role (program manager, director, clinician or other), years of experience working with youth, professional background (addictions, nursing, medicine/psychiatry, psychology, social work, child/youth work, other), and education (high school to postgraduate).

### Analysis

The data was analyzed using SPSS 24.0 [42]. Descriptive statistics were calculated to describe the characteristics of the services based on program manager or overall agency responses. Chi square tests were conducted to determine the association between agency size 207 (small/medium agency ≤30 full time staff; large agency > 30 full time staff and services) and services, referrals, and evaluation approaches. A one-way ANOVA was conducted to compare the degree of influence respondents had on agency decisions.

## Results

### Respondent and agency characteristics

Of the 869 participants, most were program managers (64.2%); other respondents included directors (9.4%); clinicians (13.6%), and respondents in other positions (2.6%); 10.1% did not describe their role at their agency. Most respondents had graduate degrees (40.5%), followed by undergraduate degrees (29.2%), college diplomas (16.5%), or other (3.2%); 10.6% did not report their education level. The median number of years respondents reported working with youth was 16 (Interquartile range [IQR] = 10–25 years). In order to gain nuance and context for their role descriptions, respondents were also asked to describe the extent to which they believed they had influence on decisions in their agencies on a visual analogue scale from 0 to 100. The perceived degree of influence was related to position, with directors (M = 78.3, SD = 15.9) reporting higher perceived influence than program managers (M = 63.1, SD = 18.9), who reported higher perceived influence than clinicians (M = 45.1, SD = 24.7; all *p value*s < .001).

**Fig. 1:**
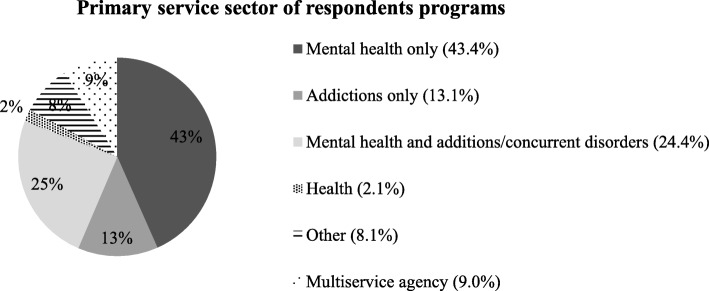
Primary service sector of respondents programs (percent)

Respondent agencies were located primarily in urban or suburban communities (68.8%), with 22.3% in rural or remote communities, and 9.0% reporting other. Almost half of reporting agencies had fewer than 30 employees (45.3%). While most respondents (63.1%) reported completing the survey based solely on their own knowledge, 19.3% consulted with other staff, 12.0% consulted administrative data, and 10.2% did not report on their sources of information. The majority of respondents came from programs within the mental health sector, with smaller percentages based in addictions, health, or multiservice respondent programs (Fig. 1).

### Child and youth population characteristics

**Fig. 2:**
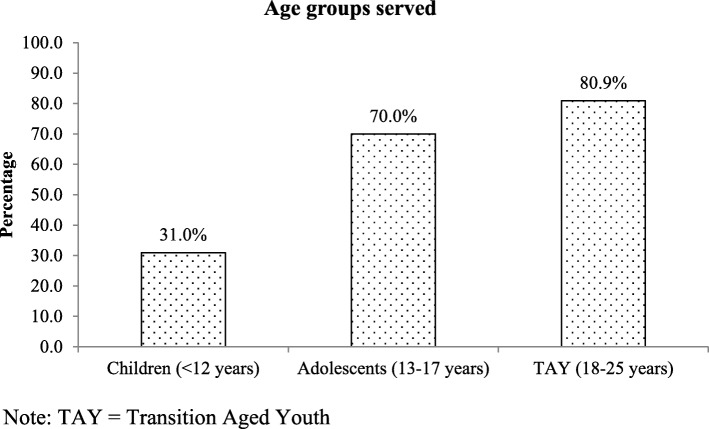
Age groups served

**Fig. 3:**
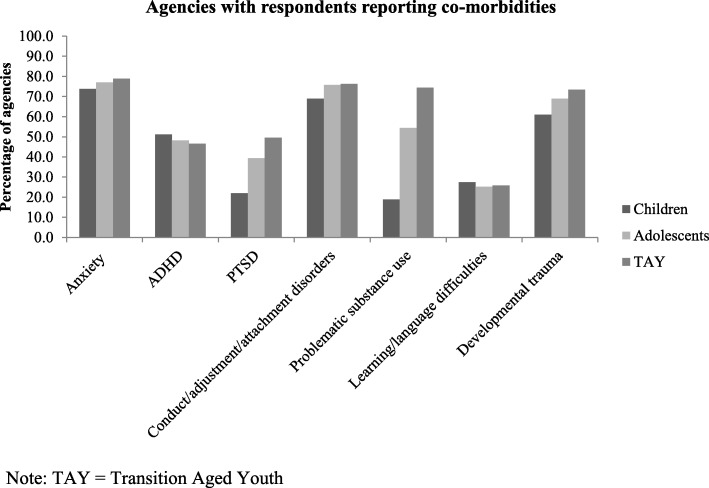
Percentage of agencies where respondents reported that more than 25% of the depressed children/youth they serve have comorbidities

In terms of populations served, a minority of respondents (31.0%) reported offering services to children under 12, while 70.0% serve adolescents between 13 and 17 years of age, and 80.9% provide services to TAY from 18 to 25 years (Fig. 2). Complexity and comorbidities were reported to be common among the depressed children and youth being served by these agencies (Fig. 3). Respondents indicated that in over 70% of agencies, more than 25% of the depressed children and youth they served also had challenges with anxiety; approximately 50% of respondents reported comorbid ADHD in at least 25% of their depressed children or youth. Both anxiety and ADHD comorbidity rates showed little variation by client age. Conversely, Post-Traumatic Stress Disorder (PTSD) and problematic substance use were reported at higher rates among agencies serving TAY, which is consistent with the known age distributions of these issues. Developmental trauma rates were reported at high rates across all age ranges (Fig. 3).

### Service characteristics

**Fig. 4:**
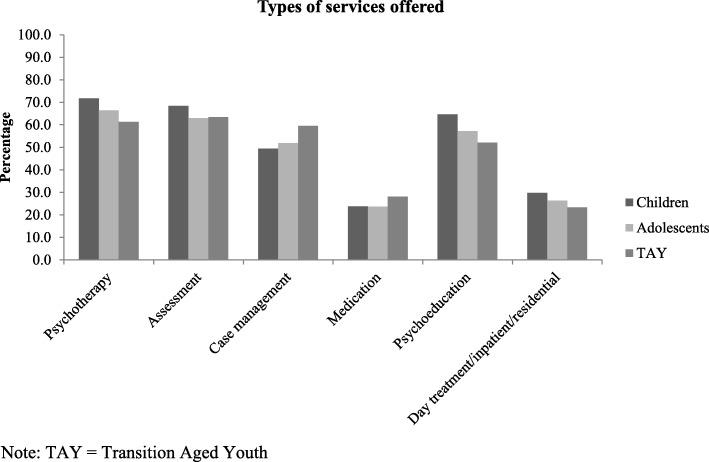
Percentage of agencies offering specific types of services, by age group

The types of services reported to be offered varied by client age (Fig. 4). Psychotherapy (61.3–71.7% of agencies) and assessment (63.0–68.4%) were the most commonly reported types of services available, followed by psychoeducation. Medication treatment was much less commonly offered (23.7–28.2%) possibly reflecting low availability of physicians to prescribe. Inpatient and residential settings were also much less commonly available at participating agencies. Psychotherapy and psychoeducation were more likely to be offered for younger age groups, while case management was more likely to be offered as a service for older youth. With the exception of medication, large agencies were significantly more likely to offer all services than smaller agencies (Table 1).

**Table 1:** Proportion of agencies offering specific services, by size of the agency^a^

Services	Small/Medium *N* = 187	Large *N* = 190	χ^2^	*p*
Psychotherapy	75.4%	89.5%	12.9	< 0.001
Assessment	65.8%	84.2%	17.1	< 0. 001
Case management	58.8%	87.9%	40.9	< 0. 001
Medication	33.7%	42.6%	3.2	0.074
Psychoeducation	62.0%	80.0%	14.8	< 0. 001
Day treatment/Inpatient/Residential	26.7%	48.4%	18.9	< 0. 001

Note: ^a^Small/medium agency ≤30 full time staff; large agency > 30 full time staff. Contradictory responses between respondents from the same agency (*N* = 36) were not included

### Availability of specific psychotherapeutic approaches

**Fig. 5:**
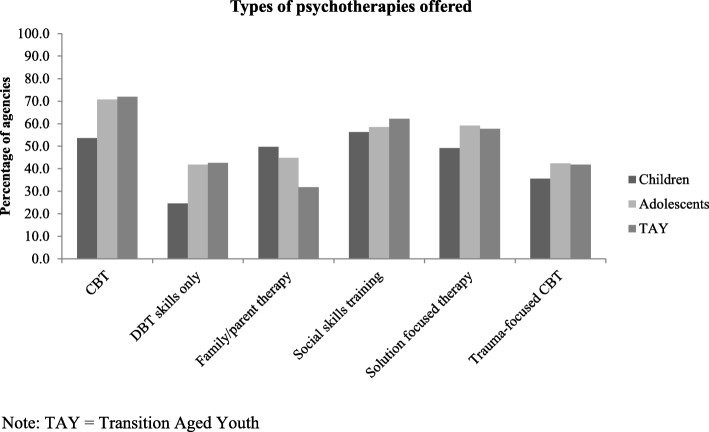
Percentage of agencies offering specific psychotherapies, by age group

A range of psychotherapies for children, adolescents and TAY were reported to be offered by respondents among participating agencies (Fig. 5). Among agencies offering services to children, “social skills training”, CBT, and family/parent therapies were most commonly reported. Among agencies offering services to adolescents and TAY, CBT, “social skills training”, and “solution-focused therapies” were most frequently reported. Reported availability of some therapies varied significantly by age group: CBT and DBT Skills were more frequently reported as available for adolescents and youth, and family/parent therapies were more frequently offered for children. Interpersonal therapy (IPT) was not among the most commonly reported therapies; among agencies offering psychotherapy to children, 10.9% reported offering IPT to children and among agencies offering psychotherapy to adolescents and TAY, 17.6–19.6% reported offering them IPT.

### Psychotherapy services: format, frequency, duration and total sessions

As can be seen in Table 2, agencies were as likely to offer individual therapy less than weekly as weekly or more frequently (Table 2). In addition, individual therapy for depression and related concerns across age groups was more likely to involve 4 or more sessions and span 3 months or more than shorter durations or fewer sessions. However, a considerable proportion of agencies (37.2–50.4%) reported offering less than 12 weeks of individual therapy, while a similar proportion reported offering a total of only 3 sessions or less (33.1–44.8%). Among agencies offering group therapy, therapy was more likely to involve 4 or more sessions and extending over 3 months, although therapy was more likely to be offered weekly or more frequently. These patterns were consistent across age groups (Table 2). Over 60% of agencies offer family therapy to children, adolescents or TAY, typically less than weekly (Table 2).

**Table 2:** Among agencies offering individual, group, and family therapy: frequency, duration, and total number of sessions

	Children (%)	Adolescents (%)	TAY (%)
Individual therapy			
Frequency			
2003Less than weekly	60.1	75.2	75.7
Weekly or more	61.1	76.8	76.2
Duration			
Less than 3 months	37.2	50.4	48.8
3 months or more	63.5	79.9	82.7
Number of total sessions			
1–3 sessions	33.1	44.8	43.4
4 or more sessions	66.9	85.1	88.4
Group therapy			
Frequency			
Less than weekly	8.1	23.1	29.4
Weekly or more	40.4	66.7	64.4
Duration			
Less than 3 months	33.0	44.9	45.9
3 months or more	38.0	62.8	66.7
Number of total sessions			
1–3 sessions	6.9	14.8	17.6
4 or more sessions	52.0	81.9	81.8
Family therapy			
Frequency			
Less than weekly	63.0	74.2	69.4
Weekly or more	46.5	47.8	35.0
Duration			
Less than 3 months	44.8	53.6	49.4
3 months or more	59.2	67.6	53.9
Number of total sessions			
1–3 sessions	42.5	56.0	55.4
4 or more sessions	59.8	65.4	50.3

Note: *TAY* Transition Aged Youth

### Service engagement

For each age group and psychotherapy type, respondents indicated their agencies’ experiences with discontinuation rates of more than 25% at each stage of service (i.e., before intake, before first session, after first session, after 2–6 sessions). Results show that no-show and discontinuation rates climb with age, with TAY generally demonstrating the highest rates (Table 3).

**Table 3:** Percentage of agencies with more than 25% no show or discontinuation rates at various stages of treatment

Age group	Individual therapy	Group therapy	Family therapy
Children	(*N* = 122)	(*N* = 60)	(*N* = 100)
No show for intake	4.9%	3.3%	2.0%
No show for first session	9.8%	6.7%	6.0%
Discontinue after first session	6.6%	10.0%	12.0%
Discontinue after 2–6 sessions	24.6%	11.7%	21.0%
Adolescents	(*N* = 240)	(*N* = 141)	(*N* = 168)
No show for intake	23.8%	16.3%	7.7%
No show for first session	25.8%	19.9%	8.3%
Discontinue after first session	22.5%	18.4%	14.9%
Discontinue after 2–6 sessions	37.9%	24.1%	23.8%
TAY	(*N* = 238)	(*N* = 138)	(*N* = 130)
No show for intake	32.8%	26.8%	10.8%
No show for first session	29.4%	20.3%	10.0%
Discontinue after first session	26.9%	18.8%	19.2%
Discontinue after 2–6 sessions	37.0%	23.9%	22.3%

*TAY* Transition aged youth

### Agency activities

Respondents reported high rates of agency referrals to other mental health services and physicians, across small/medium and large agencies. The only significant differences between small/medium and large agencies were in rates of medical doctor (MD) referrals and child welfare referrals, which were both higher in large agencies (Table 4). Outcome evaluations and quality improvement measures also varied significantly by agency size (Table 5). Respondents from larger agencies were more likely to report using client satisfaction surveys, assessing clients at the end of treatment, following-up after treatment, monitoring treatment fidelity, participating in team case conferences and engagement in outcomes research. Finally, survey respondents indicated their perceived needs around future research priorities related to services for children and youth with depression. The top three research priorities identified by respondents were effective treatment for depression (41.5%), gaps in service provision (33.8%), and improving access to therapy (31.3%).

**Table 4:** Proportion of agencies referring to further services, by size of agency^a^

Further Services	Small/Medium *N* = 179	Large *N* = 184	χ^2^	*p*
MD	79.3%	90.2%	8.4	0.004
Mental health services	83.2%	87.0%	1.0	0.320
Substance use/addictions	84.4%	89.1%	1.8	0.180
Psychotherapy (Individual/Parent)	81.6%	78.8%	0.4	0.510
Education	65.4%	74.5%	3.6	0.059
Child Welfare	62.0%	78.8%	12.3	< 0. 001

Note: ^a^Small/medium agency ≤30 full time staff; large agency > 30 full time staff

**Table 5:** Proportion of agencies using evaluation approaches, by size of agency^a^

Evaluation	Small/Medium *N* = 179	Large *N* = 184	χ^2^	*p*
Client Satisfaction Survey (Children/parents)	74.3%	91.3%	18.5	< 0. 001
Assessment at the end of treatment	50.3%	69.0%	13.3	< 0. 001
Follow-up after treatment	31.3%	47.8%	10.4	0.001
Monitor treatment fidelity	15.6%	32.1%	13.4	< 0. 001
Team case conference	59.2%	86.4%	34.0	< 0. 001
Participation in outcomes research	21.8%	35.3%	8.1	0.004

Note: ^a^Small/medium agency ≤30 full time staff; large agency > 30 full time staff

## Discussion

### Access to care

This study maps the landscape of service provision for children and youth experiencing depression and related concerns across Ontario. With regard to issues of access to care, our data reflect similar concerns to those previously raised in the literature; for instance, the bulk of treatment services are offered in urban settings, with only 1 in 5 agencies serving rural or remote communities. While this service distribution is consistent with the general population distribution across the province [43], it means that 20% of children and youth have far fewer options for seeking care. Additionally, just under half of the agencies offering services are small in size, raising questions about their capacity to offer comprehensive and timely services, particularly to a complex population of depressed children and youth.

The patient populations described in this survey reflect the challenges in the current landscape of child and youth mental health, with high rates of comorbidities, increasing prevalence and high acuity. For instance, while most reported services were targeted to adolescent and TAY, and depressive disorders do have their peak onset in these age groups, rates are rising among children, indicating a need for more child services, particularly given the data indicating that earlier age of onset predicts a more severe course of depression [44]. PTSD and problematic substance use, which both have significant impacts on treatment outcomes, were reported at higher rates among agencies serving TAY, which is consistent with the known age distributions of these issues. As is increasingly the norm [45, 46], there were high rates of complexity and comorbidities reported among depressed children and youth, increasing the acuity and worsening the prognoses for their mood disorders. Over the past 10 years in Canada, children and youth with mental disorders have had a 66% increase in visits to the emergency department, and a 55% increase in hospitalizations, putting enormous pressures on acute care services, and demonstrating a need for increased treatment services before these conditions become so severe [47].

### Service provision

Our findings show that depressed children and youth are being offered a range of services from initial assessment to treatments encompassing psychotherapy, medication and more intensive inpatient, day treatment or residential services. These are evidence-based treatments for child and youth depression when delivered with fidelity; however, evidence-based practice has proven challenging to disseminate and implement widely [28], and our data demonstrate that Ontario agencies are facing these same issues.

When compared with the NICE guidelines for the treatment of child and youth depression, the findings from this study show that the majority of agencies across Ontario do offer some of the treatments with best evidence, but with concerning features such as very low rates and inconsistent frequencies. While CBT was offered at the highest rates of any of the therapies, social skills training and solution focused psychotherapy were reported at the next-highest rates, though neither treatment is included in the NICE guidelines as best evidence for treatment of depression. IPT, which is the other evidence based individual psychotherapy outlined in the guidelines, was reportedly offered to children by 10.9% of agencies offering psychotherapy to children, and by 17.6–19.6% of agencies offering psychotherapy to adolescents and TAY. Since IPT is not designed or recommended for the treatment of childhood depression, the rates among children should be lower, while the rates among older age groups would ideally be higher. Family/parent therapies, which are evidence-based and in the NICE guidelines, were also offered at only a minority of agencies, and medication treatments were offered at fewer than 28% of agencies. While a majority of all agencies referred depressed children and youth to MDs when necessary, larger agencies were significantly more likely to refer, suggesting that moderate to severely depressed children and youth in smaller agencies may not be accessing all evidence-based treatment options.

The data on service delivery and uptake are also concerning. The evidence-based directive to offer weekly therapy for at least 3 months does not appear to be uniformly adopted, for example, as up to 40% of agencies (especially those serving children) do not offer therapy at this frequency. Additionally, the data on total number of sessions and dropout rates indicate that the quantity of therapy actually being received by depressed children and youth falls far short of the recommendations. One in five respondents reported that more than 25% of their adolescent clients either do not show up for intake or for their first treatment session. A further third of respondents reported that more than 25% of their depressed adolescents and TAY discontinue individual treatment after 2–6 sessions. No show and discontinuation rates for children appear to be lower, but still 25% of respondents reported that more than 25% of their child clients discontinue individual therapy between sessions 2 and 6. Family therapy had the fewest number of respondents reporting high drop-out rates among adolescents and TAY, but was also offered in fewer agencies and at reduced frequencies.

Thus our data reflect that the provision of evidence-based treatment of child and youth depression in Ontario is hampered both by the nature of the services being offered (treatments with low evidence being offered as well as inadequate provision of those treatments with evidence) as well as issues of treatment non-engagement. Understanding the poor rates of treatment engagement is essential to addressing the growing pressures on acute mental health care services such as emergency departments and inpatient settings. The increased demand on these services reflects an unmet earlier treatment need. Given the lifetime depression prevalence of 11% among youth, and the enduring lifelong negative impacts, successful early intervention holds the promise of significant public health benefits. The evidence supports that there are effective treatments, yet their uptake by children and youth is still partial, and their delivery formats are not consistent with best-evidence guidelines. Service providers themselves are clearly aware of these service provision issues, with the top identified research and knowledge translation priorities of effective treatment for depression, addressing service gaps and improving access to therapy.

Ongoing outcome measurement and service delivery evaluation contribute significantly to effective services [48]. Respondents reported using client satisfaction surveys as their main means of evaluation; however, these reports are less likely to include those patients who dropout mid-treatment, and also are not going to capture the experiences of clients who do not return after their first assessment meeting. 70% of larger agencies assess clients at the end of treatment, while only 50% of the smaller agencies do so. However, these assessments too would presumably not capture those clients who ended treatment before it was complete. Evaluation of the quality of the treatments being provided was also lacking, with only 15–30% of agencies taking some steps to monitor treatment fidelity. Measuring how closely the service being provided actually adheres to the principles of the modality (eg. delivering “true” CBT, rather than an approximation) is necessary when determining whether a client’s non-response is due to a failure of the treatment or of its application.

### Strengths and limitations

This study may be limited by the representativeness of the sample. Currently the database developed for this study is the only known comprehensive documentation of services for children and youth experiencing depression-related concerns in Ontario, Canada. As a result, there is no comparison available in order to estimate the representativeness of the sample. For example, this study did not collect data on the ethnicity of service users across the province, so comparisons of our findings to known population distributions of ethnic groups was not possible. Another limitation is the quality of data provided by respondents. Most respondents indicated completing the survey based on their own experiences and/or perceptions, which may be affected by a variety of biases or varying understanding of the services offered. Lastly, age boundaries vary in different services which may have resulted in services being reported as available for particular age categories which may only be available for part of that age range, and thus the results may overestimate service availability. One strength of this paper is that it contributes to the literature on the knowledge-to-action gap in the provision and uptake of depression services for children and youth. Comparing service provision to best-evidence guidelines is essential to understanding and evaluating the most widely offered depression treatments for children and youth. This dataset is the first of its kind in Canada, to our knowledge. This paper also details a process by which cross-sectoral services can be mapped and described, which is much-needed given the heterogeneous, widespread and at times disconnected nature of mental health service provision.

## Conclusion

This paper maps the diverse range of services available for child and youth depression across Ontario and provides data that estimates their uptake. Many agencies do offer evidence-based treatments, although comparison to best-evidence guidelines indicates that they may experience some implementation challenges. There are a number of future directions for research needed to increase our understanding of service provision and uptake for depressed children and youth. Notably, we need to better understand which client and treatment factors may contribute to high rates of treatment drop-out. We must also measure and address the system constraints that challenge healthcare organizations, and limit their ability to offer services that match best-evidence guidelines. In mapping the current system, this study has identified areas for focused implementation support and capacity-building activities to improve service access and optimize service delivery to children and youth with depression.

## Data Availability

The datasets used and/or analyzed during the current study are available from the corresponding author on reasonable request.

## Abbreviations

ADHD: Attention Deficit Hyperactivity Disorder
CBT: Cognitive Behavioural Therapy
CMHO: Children’s Mental Health Ontario
DBT: Dialectical Behaviour Therapy
IPT: Interpersonal Therapy
IQR: Interquartile range
MD: Medical doctor
ONCAIPS: Ontario Network of Child and Adolescent Inpatient Psychiatry Services
PTSD: Post-Traumatic Stress Disorder
TAY: Transition Aged Youth
